# High-Efficiency Frequency Doubling Blue-Laser VECSEL Based on Intracavity Beam Control

**DOI:** 10.3390/s24123913

**Published:** 2024-06-17

**Authors:** Zhuo Zhang, Jianwei Zhang, Ziye Du, Chao Chen, Yinli Zhou, Jingjing Sun, Tianjiao Liu, Jiye Zhang, Xing Zhang, Yongqiang Ning, Lijun Wang

**Affiliations:** 1 State Key Laboratory of Luminescence and Applications, Changchun Institute of Optics, Fine Mechanics and Physics, Chinese Academy of Sciences, Changchun 130033, China; zhangzhuo18@mails.ucas.ac.cn (Z.Z.); duziye22@mails.ucas.ac.cn (Z.D.); zhouyinli@ciomp.ac.cn (Y.Z.); sunjingjing21@mails.ucas.ac.cn (J.S.); liutianjiao20@mails.ucas.ac.cn (T.L.); zhangjiye@ciomp.ac.cn (J.Z.); ningyq@ciomp.ac.cn (Y.N.); wanglj@ciomp.ac.cn (L.W.); 2 University of Chinese Academy of Sciences, Beijing 100049, China; 3 ACE Photonics, No. 1759 Mingxi Road, Beihu Science and Technology Development Zone, Changchun 130102, China; zhangx@ciomp.ac.cn

**Keywords:** vertical external cavity surface-emitting laser, blue laser, cavity design, intracavity beam control, frequency conversion

## Abstract

Blue lasers are integral to a variety of applications, including marine communication, underwater resource exploration, cold laser processing, laser medicine, and beyond. Vertical external cavity surface-emitting lasers (VECSELs) have the advantages of high output power and tunable wavelength, and can output blue laser via frequency doubling. In this article, a new type of intracavity beam control external-cavity structure is introduced. The laser beam waist is effectively adjusted by intracavity beam control, and the frequency conversion efficiency is improved. A laser cavity stability analysis model was developed to investigate the impact of laser cavity lens parameters and relative positions on stability. The external resonant cavity of VECSELs utilizes two optical lenses to position the beam waist near the laser output coupling mirror and locates the frequency doubling crystal at a high optical power density position to optimize frequency conversion efficiency. The VECSEL straight external-cavity structure achieves a frequency conversion efficiency of up to 60.2% at 488 nm, yielding a blue laser output exceeding 1.3 W. The full width at half maximum of the 488 nm spectrum measures approximately 0.23 nm. This intracavity beam-controlled direct external-cavity structure effectively mitigates laser mode leakage and shows potential for the development of an efficient and compact blue laser source.

## 1. Introduction

Blue laser light finds extensive applications across industries, scientific research, and medical fields. Serving as a fundamental light source, blue lasers contribute to high-brightness and high-contrast projection systems and laser televisions [1]. They are prominently employed in optical storage, material processing, environmental monitoring, medical diagnostics, and various industrial processes [2,3,4,5,6,7]. Notably, blue laser beams offer significant advantages in processing high-reflectivity metals like copper and gold, owing to their strong absorption characteristics within this spectral range [8]. Furthermore, their low absorption coefficient in seawater enables the realization of underwater laser material processing, facilitating deep-sea equipment maintenance. Leveraging these attributes, blue lasers play pivotal roles in marine communication and underwater resource detection, serving as indispensable light sources for achieving efficient and high-quality data transmission underwater [9,10,11,12]. However, blue lasers directly output from materials usually exhibit relatively low efficiency, and high-performance blue lasers are achieved using frequency conversion techniques such as frequency doubling or triple frequency doubling.

Various methods exist for achieving blue laser output, each with distinct characteristics. Semiconductor lasers based on gallium nitride (GaN) materials can directly emit blue light with a wavelength of about 450 nm, but it is difficult to achieve high power and high beam quality blue light [13]. Another common method is to convert the near-infrared laser wavelength into blue light through using nonlinear frequency conversion technology, so as to realize the blue light laser output. This often requires the use of frequency doubling crystals, such as second harmonic generation (SHG) or third harmonic generation (THG) crystals. VECSELs have significant advantages such as high power, high beam quality, and wavelength tunability, and can achieve blue light output through efficient intracavity frequency conversion. Their flexible external-cavity structure allows frequency conversion by placing nonlinear crystals in the external cavity, which significantly expands the wavelength coverage. VECSELs exhibit stable output in ultraviolet, visible and terahertz bands through frequency conversion techniques such as frequency doubling, sum frequency and difference frequency [14,15]. In particular, intracavity frequency doubling produces a visible light band, which has been widely used in various fields. Notably, numerous reports have documented VECSELs achieving blue light emission through intracavity frequency doubling [16,17].

VECSEL has a high intracavity circulating power and can achieve efficient frequency conversion by incorporating nonlinear crystals into the cavity. The calculation formula for the second harmonic conversion efficiency of nonlinear crystals can be obtained by approximating the small signal solution of the three wave coupling equation, and then the factors affecting the frequency doubling efficiency can be analyzed [18]. By selecting and cutting nonlinear crystals, the effective length, frequency doubling coefficient, and phase matching conditions of nonlinear crystals can be determined [19]. In the near-infrared band, LBO crystals are generally chosen for frequency conversion, and the crystal size and cutting direction are selected based on the output wavelength and phase matching. The factors affecting the efficiency of frequency doubling are not only affected by the characteristics of frequency doubling crystals, but also by the power density through nonlinear crystals. The frequency doubling efficiency is directly proportional to the optical power density passing through nonlinear crystals. By adjusting the intracavity beam to reduce the waist size of the beam and improve the power density of the nonlinear crystal position light, higher-frequency doubling efficiency can be achieved.

However, the typical VECSEL frequency doubling external-cavity structure, usually V-shaped or W-shaped, may be affected by laser leakage and cause power reduction. In addition, the cavity angle of the folded cavity introduces a transverse vector for the laser output photon, which increases the photon energy and causes the output wavelength to shift to a shorter wavelength [20]. On the contrary, in the straight cavity structure, the laser oscillation is more stable, and the size of the laser beam in the cavity is gradually reduced, which can better control the output mode and waist circumference. Due to the proportional relationship between the frequency doubling efficiency and the optical power density, the frequency doubling crystal needs to be placed near the beam waist position near the surface of the gain chip, but it will hinder the pump light. By controlling the beam waist position and size in the straight cavity structure, it has the potential to achieve more efficient nonlinear frequency conversion.

This article presents an efficient frequency-doubling scheme based on VECSEL external resonant cavity beam control, achieving efficient frequency conversion through optimized beam propagation paths within the resonant cavity. Factors influencing the position of optical lenses in the cavity on stability parameters were analyzed, and a high-efficiency frequency-doubling external-cavity structure controlled by an intracavity beam was designed. This approach yielded an output of over 1.3 W at 488 nm, with a frequency doubling efficiency of 60.2%.

## 2. System Overview and Methods

**Figure 1:**
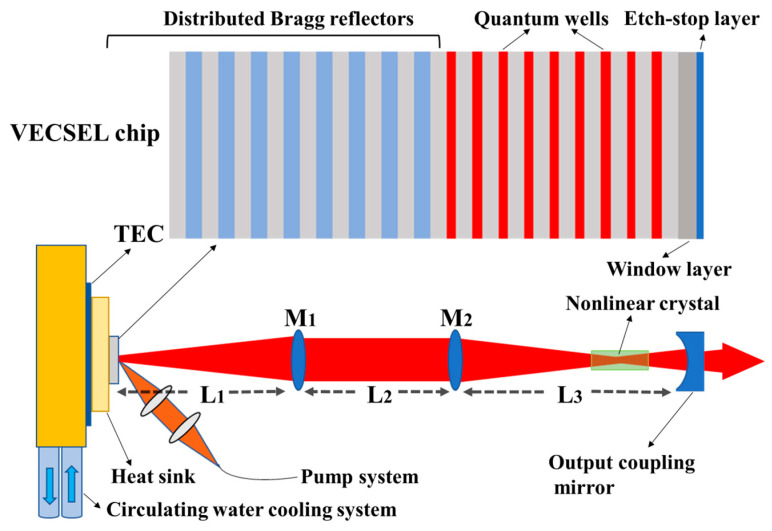
Schematic of the working principle in the high-efficient blue VECSEL.

Figure 1 shows the gain chip structure and the VECSEL external resonant cavity beam control system. The entire intracavity beam control output system follows a linear cavity VECSEL configuration, comprising an output coupling mirror, lenses M_1_ and M_2_, the gain chip, heat dissipation system, and pump system. By adjusting the intracavity lens position, beam distribution can be controlled to achieve efficient frequency conversion. The pump source system includes an 808 nm diode fiber laser with a maximum power of 100 W and a focusing lens group with a focal length of 20 mm. Adjusting the focusing mirror group allows for the accurate control of the pump spot size of the fiber output. The angle of the pump spectrum system is about 40°. The heat dissipation system consists of a thermoelectric cooler (TEC) and a water-cooling system to remove the waste heat generated by the laser during operation.

The external resonator structure consists of a gain chip and a lens M_1_, a lens M_2_ and an output coupling mirror. The output coupling mirror, coated with high reflection film in the 976 nm wavelength band and an anti-reflective film in the 488 nm band, facilitates intracavity oscillation for periodic gain and efficient frequency-doubling light output, respectively. Lenses M_1_ and M_2_ are convex lenses coated with a 980 nm band anti-reflective film to minimize reflection losses within the cavity. Additionally, the reflection film of 488 nm band is coated on the left side of M_2_, which reduces the absorption of frequency-doubling light by the gain chip and improves the output of frequency-doubling light. M_1_ adjusts the divergence angle of output light, ensuring it does not exceed the size of M_2_, while M_2_ fine-tunes the beam divergence angle, placing the beam waist between M_2_ and the output coupling mirror. The adjustment of laser cavity parameters enables flexible positioning and sizing of the beam waist in a straight cavity structure.

As shown in the illustration in Figure 1, the gain chip is designed as an optical pump structure, and utilizes a light absorption layer to absorb pump light and generate photo-generated carriers for injection into the active region. The gain chip adopts a bottom emit-ting structure [21]. During the epitaxial growth process, a buffer layer is first grown on the GaAs substrate, followed by an etching stop layer, a window layer, a multi-quantum well active region structure, and a distributed Bragg reflector (DBR). GaAs substrate removal from the gain surface, facilitated by mechanical thinning and chemical corrosion, exposes the optical port, with the GaInP etch-stop layer protecting the gain chip structure during the process.

The entire gain chip structure was epitaxially grown using the Aixtron 200/4 MOCVD system on a GaAs (100) substrate, as depicted in Figure 1. The Bragg reflector comprises 30 pairs of AlAs/GaAs material layers with a quarter-wavelength thickness, stacked in a staggered configuration, and exhibits a broad reflection band with approximately 99.9% reflectivity near the 980 nm wavelength band. The multi-quantum well active region adjacent to the Bragg reflector consists of nine 7 nanometer-thick InGaAs quantum wells, separated by GaAs barrier layers, which also serve as the pump light absorption layer. GaAsP layers flanking the quantum wells compensate for the material strain induced by InGaAs QWs [22]. A 30 nm thick AlGaAs window layer is grown on the outer side of the active region to inhibit excited state carriers from escaping to the surface and undergoing non-radiative recombination [23]. The growth process concludes with the deposition of an InGaP etching stop layer, safeguarding the integrity of the gain chip structure during substrate removal.

**Figure 2:**
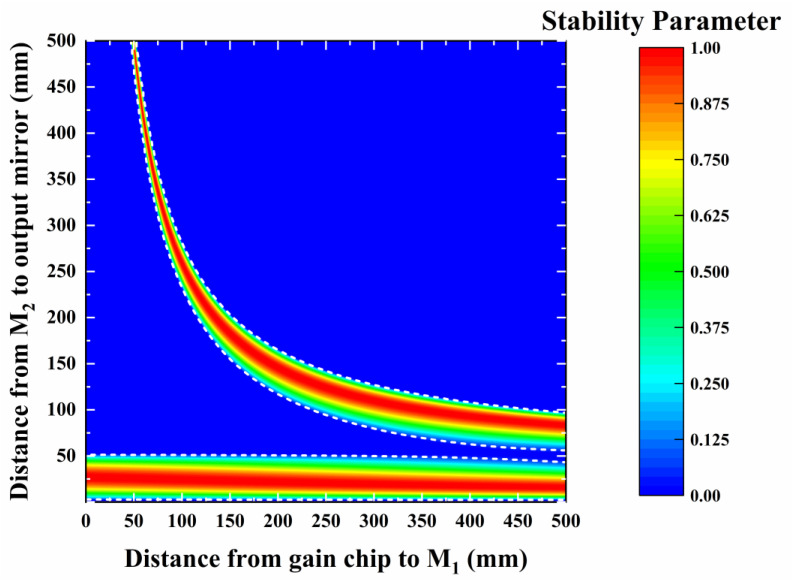
Influence of cavity lengths L_1_ and L_3_ in the VECSEL on the stability parameter in the cavity. The area enclosed by the white dotted line is the working area for cavity stabilization.

The intracavity beam control external laser cavity configuration in Figure 1 enables efficient frequency conversion, necessitating adjustments to the beam waist position and radius within the cavity. Key parameters include the distances between lens M_1_ and the chip (L_1_), between M_2_ and M_1_ (L_2_), and between M_2_ and the output coupling mirror (L_3_). M_1_ and M_2_ facilitate the adjustment of the emitted light beam’s divergence angle, while the output coupling mirror reflects the intracavity laser mode transmitted from the cavity, thereby establishing laser cavity oscillation and achieving periodic gain. A beam waist position exists between M_2_ and the output coupling mirror, allowing control over the beam distribution within the cavity by adjusting the lens position. Given the intracavity beam’s small radius, minute changes in lens position significantly impact laser cavity stability. The ABCD matrix algorithm, widely employed in laser resonator design and beam propagation analysis, efficiently analyzes the propagation of ideal beams [24,25,26]. Leveraging the VECSEL’s high beam quality and small divergence angle near the diffraction limit, we developed a theoretical model based on the generalized ABCD matrix algorithm to simulate the influence of lens parameters on laser cavity stability, facilitating a more precise laser cavity design scheme. Because the size of the gain chip is small, the position change in each lens in the laser cavity will affect the beam transmission in the cavity. Therefore, the design of the resonant cavity needs to calculate the matrix transformation of the intracavity beam ABCD matrix after passing through each lens. According to the stability condition of the coaxial spherical cavity, the stability parameter range of the resonant cavity is calculated by using the ABCD matrix [27,28]. Figure 2 shows the stable working parameter range of the laser resonator; the area where the laser resonance cannot work stably is represented by blue.

Figure 2 depicts the relationship between the distance parameters L_1_ and L_3_ in the VECSEL cavity. Two distinct stable working regions emerge within the cavity as L_1_ and L_3_ vary. In the lower stable working area, where L_3_ is below 50 mm, smaller than the curvature radius of the output coupling mirror, the waist position of the reflected and converging beam does not fall within the L_3_ range, deviating from the external resonant cavity design specifications. Conversely, in the upper stable region, where L_3_ exceeds 50 mm, the waist position of the output coupling mirror reflection convergence falls within L_3_. Notably, a stable working zone is observed within the L_3_ length range of 50 mm to 500 mm, exhibiting significant variation across different L_1_ values. The distance of L_1_ correlates with the chip’s surface beam size, typically corresponding to the pump spot size. As the VECSEL’s output power is directly proportional to the pump spot size, selecting a stable region with a broader working range in L_1_ facilitates a comprehensive analysis of beam distribution changes within the laser cavity. To mitigate transmission losses associated with prolonged cavity lengths, regions exceeding 500 mm are not considered. Opting for a stable working area where L_3_ equals 100 mm, the corresponding range of L_1_ values for a stable resonant cavity spans from 220 mm to 450 mm.

**Figure 3:**
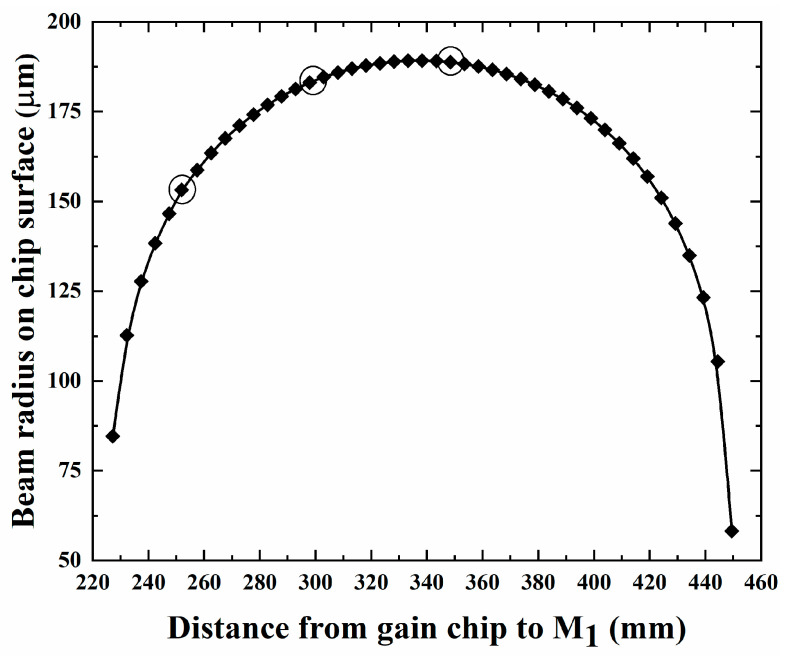
Beam radius of the cavity on the chip surface varying with the cavity length L_1_.

The cavity length L_3_ is fixed at 100 mm. Figure 3 shows the variation in the beam radius of the cavity on the chip surface with the cavity length L_1_. As the cavity length L_1_ increases, the beam radius on the chip surface initially expands and then shrinks. When the cavity length L_1_ is about 350 mm, the beam radius on the chip surface reaches its peak. With further increases in L_1_, the beam radius gradually diminishes. The diameter of the fundamental mode beam on the surface of the gain chip matches the pump spot, which can improve the output efficiency of the optically pumped laser. The larger the pump spot size matching the beam size of the gain chip surface, the higher the VECSEL output power, but there is a critical pump spot radius due to the limited heat dissipation capacity of the heat sink. Once this critical value is surpassed, the thermal resistance of the VECSEL heat sink exceeds that of the chip material, hindering effective heat dissipation within the gain chip. According to the critical value formula for pump spot size, the maximum pump spot radius supportable by a copper heat sink is approximately 200 μm [29]. At this juncture, the maximum beam radius on the gain chip’s surface in the resonant cavity measures about 187 μm, remaining within the critical pump spot size threshold of the copper heat sink.

**Figure 4:**
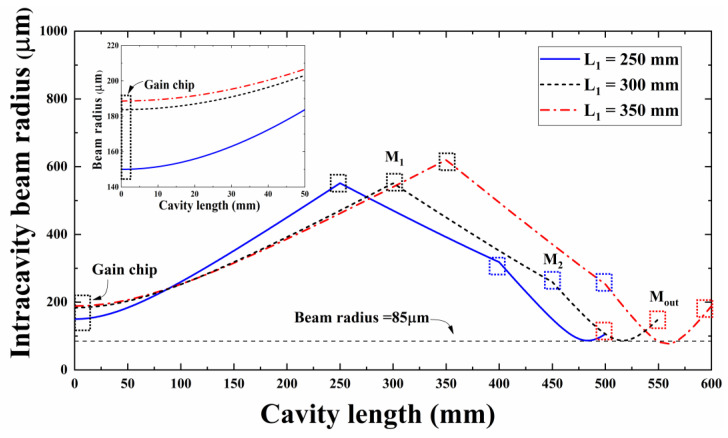
VECSEL internal oscillating laser beam distribution for L_1_ values of 250, 300, and 350 mm.

Figure 4 depicts the radius variation in beam propagation across the entire cavity at L_1_ distances of 250, 300, and 350 mm, with the positions of the cavity components illustrated. The gain chip is situated on the far-left side of the resonant cavity, denoted by a black rectangular box. The gain chip is located on the far-left side of the resonant cavity and is represented by a black rectangular frame. From the gain chip to the right, the lens M_1_ (black square box), the lens M_2_ (blue square box) and the output coupling mirror M_out_ (red square box) are in turn. The cavity lengths L_2_ and L_3_ remain unchanged. With the increase in the cavity length L_1_, the beam distribution shape in the resonant cavity does not change significantly. This observation shows the relative stability of the overall beam propagation, which is consistent with the simulation results of the stability parameters described in Figure 2. As illustrated in Figure 4, the increase in L_1_ also correlates with an augmentation in the beam radius on the surface of the gain chip. The maximum beam radius on the gain chip’s surface is attained at L_1_ = 350 mm, consistent with the simulation findings in Figure 3. The beam distribution within the resonant cavity reveals that the beam waist position lies between M_2_ and the output coupling mirror M_out_. At L_1_ = 350 mm, the beam waist position is minimal. Consequently, selecting an external-cavity structure with L_1_ equal to 350 mm enables the simultaneous attainment of a larger gain chip surface beam size and a smaller beam waist size.

## 3. Results

**Figure 5:**
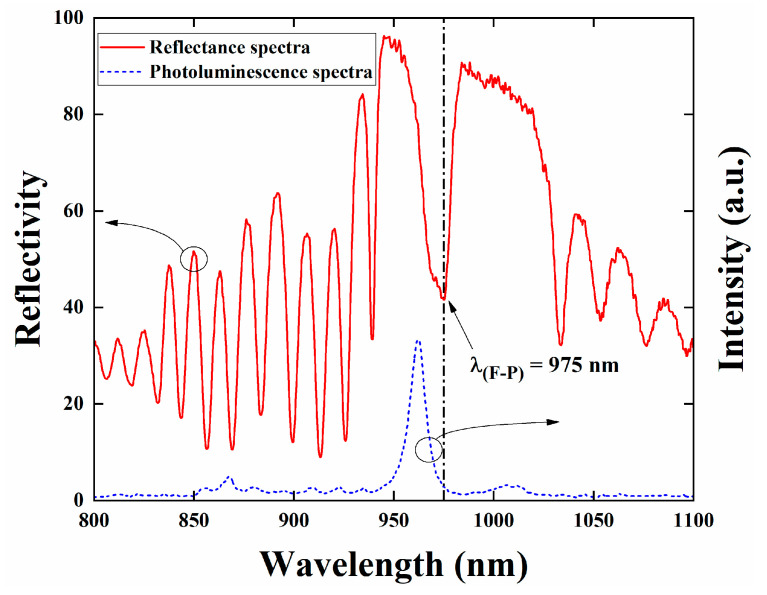
Gain chip reflection spectrum at 20 °C and photoluminescence spectra obtained at the edge and plane of the gain chip at 20 °C.

We optimized the parameters of a high-efficiency frequency doubling system utilizing intracavity beam control within an external resonant cavity through numerical simulations. Intracavity beam control narrowed the waist size of the intracavity beam and adjusted its position near the output coupling mirror, as depicted in Figure 1. Prior to conducting system performance testing, we conducted basic characteristic tests on the prepared VECSEL chip. These tests included photoluminescence spectra to represent the characteristics of the active region gain and white light reflection spectra to represent the DBR reflection characteristics, as illustrated in Figure 5. The reflection spectrum of the gain chip exhibits a distinct wide reflection band, spanning from 940 nm to 1020 nm, approximately 80 nm wide. A clear depression at the center of the reflection band signifies the resonance wavelength position of the Fabry–Perot (F-P) cavity of the gain chip. The center wavelength of the depression, at 975 nm, represents the output wavelength of the gain chip [30]. The photoluminescence spectrum, measured on the front side of the gain chip, displays a distinct gain peak without any side peaks, indicating that the strain-compensated gain chip structure exhibits good epitaxial growth quality and does not produce significant growth defects. The gain peak wavelength is situated on the left side of the cavity mode depression. The gain spectrum of the VECSEL is wide, and various laser modes oscillate in the F-P cavity to obtain periodic gain. Laser modes that do not match the F-P cavity mode experience large oscillation losses in the cavity, while modes that match the F-P cavity mode have the smallest oscillation losses. The temperature drift coefficient of the gain peak differs from that of the cavity mode, with the drift rate of the gain peak being about three times that of the cavity mode [21]. Therefore, the VECSEL structure is designed with a blue shift of the gain peak, resulting in an initial detuning from the cavity mode position. This design aims to achieve optimal matching between the gain peak and the cavity mode at high pump power, thereby achieving a high-power output and a high-slope efficiency. When the pump power is low, the internal temperature of the chip is not high enough, resulting in a large detuning between the gain peak and the F-P cavity mode. The loss generated in the cavity is converted into heat in the form of non-radiative transitions, which gradually increases the chip temperature. As the pump power increases, the internal temperature of the gain chip rises, and the temperature drift rate of the gain peak is greater than that of the F-P cavity mode. This results in the matching of the gain peak with the F-P cavity mode, reducing the oscillation loss of the laser mode in the cavity and thereby achieving higher power output. As the pump power continues to increase, the heat sink cannot dissipate the waste heat generated by the chip in time, resulting in an excessively high chip temperature and an excessive red shift in the gain peak, causing it to deviate from the F-P cavity mode again.

**Figure 6:**
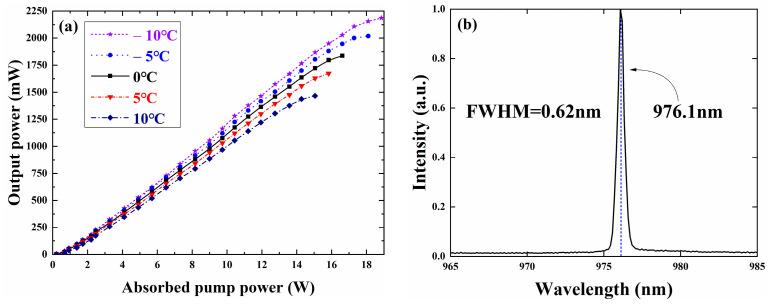
(**a**) The fundamental frequency light output power curve of VECSEL at different temperatures, (**b**) laser spectra of VECSEL at fundamental frequency light output at −10 °C.

Figure 6a shows the relationship between output power and absorbed pump power under TEC control at various temperatures. The power curves at different operating temperatures exhibit similar trends of variation. As the absorbed pump power increases, the output power of the VECSEL shows a linear increment. However, upon reaching the critical pump power, excessive heat generation by the gain chip surpasses the heat dissipation capacity of the heat sink. Consequently, the temperature of the active region escalates rapidly, leading to heightened non-radiative transitions and a decrease in the slope efficiency of the power curve. The temperature drift coefficient of the gain peak is larger than that of the cavity mode. As the pump power increases, the internal temperature of the chip rises, and the red shift of the gain peak exceeds that of the cavity mode. There is a detuning of about 12 nm between the gain peak and the cavity mode. The higher the internal temperature of the chip, the better the matching between the gain peak and the cavity mode, thus reducing the oscillation loss of the laser mode in the cavity and improving the slope efficiency of the laser. However, the excessively high operating temperature of the gain chip will result in an excessive red shift of the gain peak, causing it to shift to a longer wavelength position than the cavity mode. This leads to a significant mismatch between the gain peak and the cavity mode. At high pump power, the gain peak-cavity mode mismatch results in a more serious accumulation of waste heat generated by non-radiative transitions, triggering the thermal flip phenomenon and leading to a decrease in output power. Conversely, as the working temperature controlled by TEC decreases, the maximum output power of the VECSEL gradually increases. This rise is attributed to the heightened rate of heat dissipation by the radiator, necessitating a higher pump power for thermal inversion. The maximum output power of VECSEL is more than 2.186 W at 976 nm. In Figure 6b, the laser spectrum of the VECSEL fundamental frequency light output at 0 °C is depicted. The center wavelength of the laser spectrum measures 976.1 nm, with a full width at half height of 0.62 nm.

**Figure 7:**
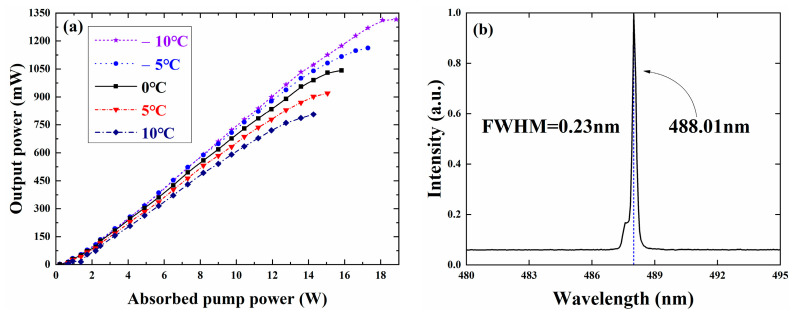
(**a**) The output power curves of VECSEL at different temperatures for frequency-doubling light, and (**b**) the laser spectrum of VECSEL under frequency-doubling light output at −10 °C.

According to the formula derived from the nonlinear transformation theory, the beam waist radius is about 70 μm, indicating that the optimal length of the LBO crystal is about 7 mm [31]. Therefore, we chose a 3 mm × 3 mm × 7 mm LBO crystal for frequency doubling and positioned it near the waist of the output coupling mirror, as shown in Figure 1. Figure 7a describes the relationship between the output power of frequency-doubling light and the absorbed pump power under TEC control at different temperatures. The power curve of the frequency-doubling light shows a similar trend with the power curve of the fundamental light in Figure 6a. The critical absorption pump power corresponding to the thermal flip of the doubling frequency light power curve is almost consistent with the fundamental frequency light, indicating a good stability of the external-cavity structure. The placement of LBO in the resonant cavity hardly increases additional cavity losses. Furthermore, the maximum output power of VECSEL 488 nm doubled light reaches 1.316 W, with a conversion efficiency between fundamental and doubled light of 60.2%. In Figure 7b, the laser spectrum of VECSEL frequency-doubling light output at −10 °C is presented, revealing a center wavelength of 488.01 nm and a full width at half height of 0.23 nm.

**Figure 8:**
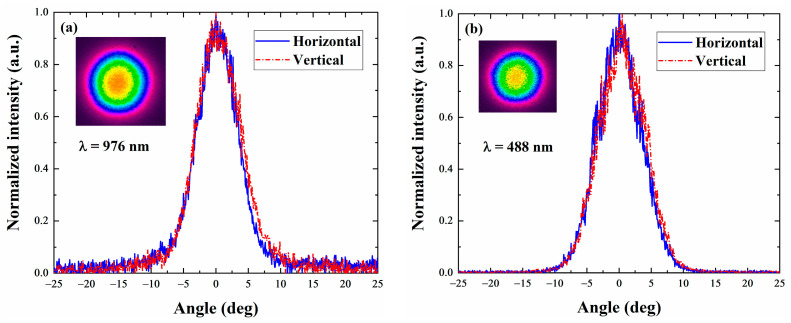
One-dimensional far-field mode of VECSEL at (**a**) 976 nm fundamental frequency light and (**b**) 488 nm doubling frequency light output. The VECSEL beam profile is shown in the illustration.

Figure 8 shows the far-field mode distribution of the 976 nm fundamental frequency light and the 488 nm doubling frequency light of VECSEL at an operating temperature of −10 °C. The diagram presents the intensity distribution profile of the 2D beam captured using a charge-coupled device (CCD). The spot morphology shows a good circularly symmetric Gaussian beam shape and maintains a circularly symmetric distribution in two dimensions. The divergence angle of the 976 nm fundamental light is 7.5°, while the divergence angle of the 488 nm fundamental light is 7.3°.

## 4. Discussion

Based on the distinctive external-cavity structure of VECSEL, we devised a cavity beam-controlled external resonant cavity configuration capable of efficient frequency conversion. Employing the generalized ABCD transfer matrix for beam transmission analysis within the laser cavity, we determined the stability parameters based on coaxial spherical cavity stability conditions. Subsequently, an external-cavity structure was engineered to regulate beam distribution within the cavity using cavity lenses. By adjusting the parameters of the laser intracavity lens, a broad stable region was achieved within the laser cavity, with the beam waist positioned near the output coupling mirror. This adjustment increased the size of the intracavity beam fundamental-mode spot on the gain chip surface to support a larger pump spot and enable high-power output. This high-efficiency frequency-doubling external cavity scheme achieved a conversion rate of up to 60% between fundamental frequency light and frequency-doubling light at −10 degrees Celsius, producing over 1.3 W output for 488 nm frequency-doubling light, with a full width at half height of 0.23 nm. The beam profiles of VECSEL output at a 976 nm fundamental frequency and a 488 nm doubling frequency exhibit a Gaussian distribution, with a divergence angle of less than 7.5°.

The unique external-cavity structure of optically pumped surface-emitting semiconductor lasers facilitates the integration of optical components for various operations, including line-width narrowing, frequency conversion, wavelength tuning, and mode locking. The proposed high-efficiency frequency conversion scheme for a straight laser resonant cavity based on the VECSEL intracavity beam control offers greater stability compared to the V-shaped external cavity system structure, enabling the precise control of output modes without causing laser leakage. However, some challenges persist with the current intracavity beam control and external-cavity structure, notably a high intracavity loss resulting in lower overall output power. Future endeavors will focus on optimizing the intracavity beam control system to achieve high-power and high-efficiency frequency conversion, thus delivering high-performance blue light sources for applications such as marine resource exploration and underwater communication.

## Data Availability

Experimental data are available upon reasonable request to the authors.
